# Effects of Left Ventricular Wall Motion Abnormality on Global and Regional Diastolic Function of the Left and Right Ventricles at Rest and After Stress

**DOI:** 10.14740/cr366w

**Published:** 2014-12-04

**Authors:** Dawod Sharif, Amal Sharif-Rasslan, Majed Odeh, Camilia Shahla, Amin Khalil, Uri Rosenschien

**Affiliations:** a Department of Cardiology, Bnai Zion Medical Center, Haifa, Israel; b Technion - Israel Institute of Technology, Haifa, Israel; c Mathematics Department, The Academic Arab College, Haifa, Israel; d Department of Internal Medicine A, Bnai Zion Medical Center, Haifa, Israel

**Keywords:** Diastolic function, Left ventricle, Right ventricle, Dobutamine stress echocardiography, Ischemia

## Abstract

**Background:**

Diastolic dysfunction precedes systolic dysfunction in patients with coronary artery disease. The aim of the study was to evaluate the effects of left ventricular (LV) wall motion abnormality (WMA) on diastolic LV and right ventricular (RV) function at rest and after stress.

**Methods:**

Fifty-nine subjects, 15 with LV-WMA (abnormal group) and 44 without (normal group), underwent dobutamine stress echocardiography (DSE) studies, in addition to evaluation of LV and RV diastolic function before and after DSE.

**Results:**

Resting mitral flow parameters were similar. DSE increased peak A-wave velocities in both groups, and mitral color slope only in normal subjects. After DSE, E-wave peak velocities and mitral color slope were higher in normal subjects, P < 0.05. At rest and after DSE systolic and diastolic pulmonary vein velocities were similar in both groups; however, DSE increased these velocities only in normal subjects, P < 0.05. Regional E-wave peak velocities of LV were higher at rest in normal subjects, P < 0.05. Both LV and RV, regional peak E-wave velocities were not affected by DSE. After DSE, regional A-wave peak velocities increased in all (P < 0.01), except at the lateral region (P = 0.07). DSE increased trans-tricuspid velocities in both groups, P < 0.05. Resting A-wave velocities were higher in normal subjects, P < 0.01.

**Conclusions:**

Global LV early diastolic filling parameters were not affected by LV-WMA at rest. LV-WMA blunted the response after stress. RV E-wave velocities increased after DSE, and were not affected by LV-WMA. LV-WMA reduced regional LV-E’ velocities at rest but not the reserve. A-wave velocities were not affected by WMA and increased after DSE.

## Introduction

Myocardial fibers of both ventricles have similar helical architecture and are linked to each other [1, 2]. Dynamics of systolic left ventricular (LV) and right ventricular (RV) longitudinal performance and reserve have been described and shown to be linked [3, 4].

Longitudinal ventricular systolic dysfunction precedes radial abnormalities [5]. According to the ischemic cascade, diastolic ventricular dysfunction precedes both radial and longitudinal systolic dysfunction [6, 7]. Regional ventricular dysfunction also precedes global ventricular dysfunction. Moreover, symptoms on effort precede complaints at rest. Therefore, the aim of this prospective study was to evaluate changes and link between regional LV and RV diastolic function on dobutamine stress echocardiography (DSE).

## Methods

### Population

Fifty-nine patients without the exclusion criteria, 29 women, aged 60.3 ± 13.4 years, were prospectively evaluated for the presence of coronary artery disease. Fifteen of the subjects (abnormal group) had wall motion abnormality (WMA) at rest, and 44 had no WMA at rest (normal group). Exclusion criteria included presence of pulmonary disease, right heart disease, valvular heart disease, atrial fibrillation or other rhythm disturbances and acute coronary syndrome within the last 3 months were not included. All subjects underwent DSE studies using the usual protocol, and measurement of mitral and tricuspid diastolic annular velocities before and immediately after the stress study.

### DSE

The protocol of dobutamine infusion consisted of 3 min stages for each dose, staring with 5 µg/kg/min and increasing to 10, 20, 30 and 40 µg/kg/min. If end-points did not occur or 85% of the age adjusted heart rate was not achieved, 0.25 mg atropine was injected every 2 min up to 1 mg or until the target heart rate was achieved. Blood pressure and 12-lead electrocardiograms were recorded at rest and throughout the DSE study. Horizontal or down-sloping > 1 mm ST-segment depression at 0.06 s after the J point were considered as evidence for myocardial ischemia.

### Image acquisition

Images were obtained while the patients in the left lateral decubitus position. A standard commercial Siemens, Acuson Sequoia echocardiography system, California, equipped with 3.5 MHz transducers was used. All patients had complete Doppler echocardiographic studies before DSE studies. Parasternal long axis and short axis as well as apical four-chamber and two-chamber views were recorded at rest, low dose dobutamine infusion, peak exercise and in the recovery period. Digital images were stored on magneto-optic discs for later off-line analysis. In addition, super VHS videotape recordings were performed throughout the studies.

### DSE study analysis

**Figure 1:**
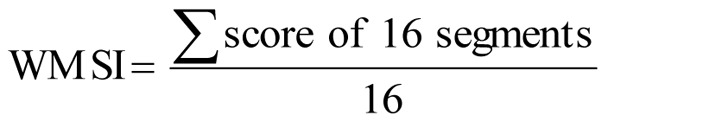
Calculation of wall motion score index.

Segmental LV wall motion analysis was performed using 16-segment model. Regional wall motion and segmental score was estimated as normal = 1, hypokinetic = 2, akinetic = 3 or dyskinetic = 4. New or worsening segmental WMA was considered as ischemic response. Ischemic response (I) was identified when wall motion decreased by at least one grade in two adjacent segments or wall motion decreased by at least two grades in one segment, otherwise no ischemia, or normal response (N) was diagnosed. Wall motion score index (WMSI) was calculated as in Figure 1.

### Acquisition of ventricular inflow velocities

The sample volume of the pulsed wave Doppler was placed at the tips of mitral and tricuspid valves to record LV and RV inflow velocities. At rest, easily discernible E and A wave velocities were recorded in all subjects. After DSE studies, recording of ventricular inflow velocities was delayed until the earliest separation of E and A waves was observed at heart rates 108 ± 2 bpm as previously reported [8]. Measurements of peak E and A velocities as well as E-deceleration times were averaged from three consecutive beats. Measurements of the slopes of color Doppler-M ventricular inflow tracings were measured from three consecutive beats.

### Pulmonary venous velocities

Pulmonary venous peak systolic and diastolic velocities were measured form the apical four-chamber view and averaged from three consecutive beats.

### Tissue Doppler imaging

The apical four-chamber, two-chamber and three-chamber views were used to assess the longitudinal diastolic velocities of the mitral annulus. The sample volume of the pulsed wave Doppler was located at the mitral annulus and recording was performed from the septal, lateral, anterior, inferior and posterior portions of the annulus. In addition, Doppler sampling from the proximal anteroseptal segment was performed. Tricuspid diastolic annular velocities were recorded from the lateral border from the apical four-chamber view. Annular Doppler velocities were recorded on videotape for off-line analysis. Measurements were averaged from three consecutive beats.

### Statistical analysis

Statistical analyses were conducted using SPSS software version 13 (IBM, Petach-Tikva, Israel). Mean values and standard deviation of the six annular mitral and the lateral tricuspid annular longitudinal diastolic velocities were calculated for all. Student’s *t*-test assuming unequal variances was used to compare the normal and abnormal groups, while paired *t*-test was performed to compare the parameters before and after DSE in both groups; P < 0.05 was considered significant.

## Results

All subjects underwent DSE studies safely and uneventfully. Heart rate increased from 62.4 ± 9.9 bpm to 133 ± 14.4 bpm. Systolic blood pressure increased from 138 ± 7 mm Hg to 162 ± 9 mm Hg. In the normal group, heart rate increased from 70.7 ± 10 bpm to 138 ± 11.8 bpm, P < 0.00001, and in the abnormal group, heart rate increased from 74.2 ± 17.6 bpm to 130.9 ± 15.6 bpm, P < 0.00001. Heart rate was similar between groups both at rest and after DSE studies. Systolic blood pressure increased from 138 ± 7 mm Hg to 162 ± 9 mm Hg, P < 0.0001, in all subjects without significant inter-group difference.

### LV wall motion analysis

In 26 subjects, WMSI was 1 at rest and did not change after DSE, while in 18 subjects WMSI was 1 at rest and increased to 1.12 ± 0.14 after stress. In 15 subjects, WMSI at rest was 1.36 ± 0.34 and increased to 1.47 ± 0.32 after DSE.

### LV filling parameters

#### Global LV filling parameters at rest

At rest, no significant difference was found in trans-mitral filling pulsed-wave Doppler velocity parameters, mitral color slope or pulsed-wave pulmonary venous velocities between groups with and without LV-WMA (Table 1).

**Table 1:** Global Left Ventricular Diastolic Filling Parameters at Rest and After DSE

		MV-E (cm/s)	MV-A (cm/s)	MV-E/A	EDT (ms)	MV-CS (cm/s)	PV-D (cm/s)	PV-S (cm/s)	PV-D/S
Rest	Abnormal	69 ± 22	78 ± 17	0.93 ± 0.42	205 ± 74.1	50.5 ± 12.6	46.8 ± 13.8	54.4 ± 14.75	0.83 ± 0.475
	Normal	79 ± 15	77.9 ± 16.5	1.02 ± 0.25	165.4 ± 30.5	49.45 ± 12.2	51.76 ± 12.37	61.2 ± 13.55	0.887 ± 0.295
	P	0.16	0.6	0.517	0.215	0.82	0.274	0.173	0.653
DSE	Abnormal	65 ± 0.12	103 ± 0.15	0.64 ± 0.13		50.4 ± 13.2	58.1 ± 22.6	69.4 ± 33.71	0.361 ± 1.357
	Normal	83 ± 0.17	111 ± 0.27	0.79 ± 0.23		62.5 ± 15.2	63.07 ± 20.35	87.15 ± 20.9	0.758 ± 0.348
	P	0.0014	0.22	0.017		0.0224	0.537	0.14	0.382
P (rest:DSE)	Abnormal	0.558	0.0015	0.0296		0.982	0.187	0.216	0.215
	Normal	0.222	6.37 × 10^-7^	9.67 × 10^-5^		5.83 × 10^-5^	0.0038	7.81 × 10^-9^	0.079

A: late diastolic A-wave peak velocity; E: early diastolic mitral E-wave peak velocity; MV: mitral valve; CS: early diastolic color slope; EDT: mitral E-wave deceleration time; PV: pulmonary vein; D: peak diastolic velocity; S: peak systolic velocity.

#### Effects of DSE on global LV filling parameters

In both groups, after DSE studies, peak E-wave velocity did not increase significantly. Mitral early diastolic color Doppler slope increased only in the normal group (Table 1). Peak late diastolic A-wave velocity increased in both groups. As a result, the ratio of E/A peak velocity decreased in both groups after DSE studies (Table 1). After DSE, pulmonary venous diastolic and systolic velocities increased only in the normal group and were associated with decrease in their ratio.

#### Global LV filling parameters after DSE

After DSE studies, the normal group had faster early diastolic LV filling with higher peak E-wave velocity, peak E/A velocity ratio and faster early diastolic mitral color Doppler slope compared to the abnormal group (Table 1). Pulmonary venous velocities were similar in both groups (Table 1).

#### Regional LV tissue-Doppler velocities

Annular E and A velocities were discernable in all subjects both at rest and after DSE studies.

#### Regional LV diastolic velocities at rest

At rest, LV tissue-Doppler early diastolic E’ velocities were higher in all proximal regions in the normal group compared to the abnormal group (Table 2). Late diastolic A’ velocities were similar in both groups (Table 2). E/E’ and A/A’ velocity ratios at rest were similar in both groups (Table 3).

**Table 2:** Regional Left Ventricular Diastolic Tissue Doppler Parameters at Rest and After Stress

		E_IVS_ (cm/s)	E_Lat_ (cm/s)	E_inf_ (cm/s)	E_ant_ (cm/s)	E_posterior_ (cm/s)	E_RV_ (cm/s)	A_IVS_ (cm/s)	A_Lat_ (cm/s)	A_inf_ (cm/s)	A_ant_ (cm/s)	A_posterior_ (cm/s)	A_RV_ (cm/s)
Rest	Abnormal	9.63 ± 2.57	11 ± 3.1	9.34 ± 2.6	10.95 ± 2.96	10.2 ± 2.3	16.79 ± 7.15	12.2 ± 2.8	12.9 ± 4.6	13.3 ± 2.3	14.4 ± 5.2	14.1 ± 2.19	19.4 ± 5.3
	Normal	11.9 ± 3.8	14 ± 3.8	13 ± 3.7	13.6 ± 3.8	12.4 ± 3.2	16.3 ± 3.39	12.9 ± 2.4	13.9 ± 2.64	14.6 ± 3.1	14 ± 3.5	14.7 ± 3.3	20.1 ± 6.1
	P	0.0226	0.01	0.001456	0.02779	0.02476	0.8403	0.429	0.488	0.147	0.805	0.483	0.74
DSE	Abnormal	12.1 ± 3.2	14.2 ± 4.5	10.3 ± 1.98	13.1 ± 5.25	11.2 ± 3.1	17.44 ± 8.6	17.2 ± 4.5	17 ± 5.8	19.6 ± 3.7	20.4 ± 6.4	18.4 ± 4	26.7 ± 4.1
	Normal	12.6 ± 3.6	15.7 ± 4.1	13.4 ± 3.7	14.5 ± 5.1	13.3 ± 3.9	17.02 ± 5.92	17.7 ± 4.1	17 ± 3.4	19.1 ± 3.6	18.1 ± 5.7	19.5 ± 4.8	28.3 ± 6.9
	P	0.665	0.33	0.001012	0.445	0.0879	0.884	0.768	0.96	0.733	0.338	0.474	0.36
P (rest:DSE)	Abnormal	0.0558	0.064	0.335	0.289	0.441	0.853	0.0074	0.072	0.00036	0.0417	0.00981	0.005
	Normal	0.37	0.058	0.665	0.383	0.2686	0.503	1.69 × 10^-8^	3.9 × 10^-5^	7.29 × 10^-8^	0.00033	2.02 × 10^-6^	2.4 × 10^-7^

A: late diastolic A-wave peak velocity; E: early diastolic mitral E-wave peak velocity; ant: anterior; inf: inferior; IVS: inter-ventricular septum; Lat: lateral; RV: right ventricle.

**Table 3:** Ratio of Early and Late Diastolic Mitral Peak Velocities to Regional Left Ventricular Doppler Diastolic Peak Velocities at Rest and After Stress

		MV-E/E_IVS_	MV-E/E_Lat_	MV-E/E_inf_	MVE/Eant	MV-E/E_posterior_	MV-E/E_RV_	MV-A/A_IVS_	MV-A/A_Lat_	MV-A/A_inf_	MVA/A_ant_	MV-A/A_posterrior_	MV-A/A_RV_
Rest	Abnormal	7 ± 1.9	5.7 ± 1.8	7 ± 2	6 ± 1.5	6 ± 1.7	4.48 ± 1.9	6.4 ± 0.5	5.7 ± 1.6	5.8 ± 1.2	5.7 ± 1.5	5.5 ± 1.5	0.04 ± 0.12
	Normal	7 ± 3	6 ± 2	6.7 ± 2.6	6.3 ± 2.3	7 ± 2.4	4.89 ± 1.5	6.5 ± 1.3	6 ± 2	5.8 ± 2.2	6.2 ± 2.2	5.8 ± 2	0.045 ± 0.019
	P	0.59	0.576	0.66	0.547	0.414	0.369	0.735	0.48	0.884	0.434	0.512	0.805
DSE	Abnormal	5.8 ± 2	4.9 ± 1.4	2.5 ± 0. 7	6 ± 2	6.3 ± 1.5	5.1 ± 2.5	6.5 ± 2.2	6.6 ± 1.6	6 ± 2	6.5 ± 2	6 ± 2.2	0.039 ± 0.006
	Normal	7 ± 3	5.6 ± 1.9	3 ± 1.3	6.3 ± 2.4	6.6 ± 2.6	5.49 ± 2.5	6.4 ± 1.5	6 ± 1.5	5.4 ± 1.5	5.4 ± 1.6	5.9 ± 1.3	0.04 ± 0.017
	P	0.0795	0.207	0.122	0.47	0.63	0.375	0.93	0.326	0.29	0.122	0.77	0.46
P	Abnormal	0.147	0.25	2.93 × 10^-5^	0.823	0.903	0.619	0.947	0.549	0.57	0.749	0.513	0.33
(rest:DSE)	Normal	0.83	0.32	5.15 × 10^-11^	0.96	0.57	0.482	0.865	0.269	0.85	0.575	0.663	0.41

Abbreviations as in Tables 1 and 2.

#### Effects of DSE test on regional LV diastolic velocities

DSE test significantly increased A’ velocities in all regions, while E’ velocities did not change (Table 2). After DSE test, E/E’ velocity ratios decreased significantly compared to values at rest in both groups in the inferior wall, while all the other parameters at all regions did not change significantly (Table 3).

#### Regional LV diastolic velocities after DSE test

After DSE test, only in the proximal inferior LV segment, E’ was higher in the normal group than in the abnormal group, while all the other velocities and velocity ratios were similar in both groups (Table 2).

### RV filling parameters

#### Inter-group comparison at rest

At rest trans-tricuspid late diastolic A-wave peak velocity was higher in the normal group than in the abnormal group, while the other parameters were similar (Table 4).

**Table 4:** Right Ventricular Global and Regional Diastolic Peak Velocities and Ratios at Rest and After Stress

		TV-E (cm/s)	TV-A (cm/s)	TV-E/A	E_RV_ (cm/s)	A_RV_ (cm/s)	TV-E/E_RV_	TV-E/E_IVS_	TV-A/A_RV_	TV-A/A_IVS_
Rest	Abnormal	49.8 ± 17	44.9 ± 10	1.15 ± 0.38	16.79 ± 7.15	19.4 ± 5.3	4 ± 2.8	5.8 ± 1.4	4 ± 1.2	3.85 ± 1.08
	Normal	57 ± 13	57.7 ± 14	1.04 ± 0.296	16.3 ± 3.9	20.1 ± 6.1	3.6 ± 0.01	5.3 ± 2.2	4.5 ± 1.9	4.53 ± 1.46
	P	0.27	0.01	0.466	0.8403	0.74	0.72	0.432	0.805	0.186
DSE	Abnormal	70 ± 15	77.9 ± 226	0.94 ± 0.21	17.4 ± 8.6	26.7 ± 4.1	5.6 ± 2.8	6.33 ± 1.27	3.9 ± 0.6	4.59 ± 0.93
	Normal	65 ± 12	82.4 ± 21	0.84 ± 0.24	17 ± 5.9	28.3 ± 6.8	4.4 ± 1.8	5.6 ± 2.4	4 ± 1.7	4.45 ± 1.29
	P	0.45	0.623	0.26	0.884	0.36	0.24	0.25	0.46	0.774
P (rest:DSE)	Abnormal	0.022	0.004	0.198	0.853	0.005	0.23	0.427	0.33	0.173
	Normal	0.0168	1.15 × 10^-5^	0.0069	0.503	2.4 × 10^-7^	0.046	0.6212	0.41	0.859

TV: tricuspid valve; other abbreviations like in previous tables.

#### Effects of DSE test

DSE test increased peak E-wave and A-wave tricuspid velocities in both groups; however, E/A velocity ratio decreased only in the normal group (Table 4). While A’ peak velocity increased in both groups, peak E’ velocity of RV free wall did not change (Table 4). The ratio of E/E’ RV peak velocities increased only in the normal group after DSE test (Table 4).

#### Inter-group comparison after DSE test

After DSE test, no difference in any parameter was observed between the groups (Table 4).

## Discussion

Global early diastolic LV filling velocities at rest, as assessed by mitral E-wave velocity, mitral color slope and pulmonary venous velocities, were not affected by the presence of LV-WMA. On the other hand, regional early diastolic velocities by tissue Doppler were lower in the presence of LV-WMA. Global LV early diastolic E-wave peak velocity after DSE test was higher in the absence of WMA. In the normal group, a marked increase in global early LV filling parameters and pulmonary venous return were observed after DSE test, which may indicate a better diastolic reserve and enhanced LV diastolic relaxation. Late diastolic global and regional A-wave velocities increased after DSE test and were not affected by LV-WMA both at rest and after stress. The ratio of mitral E/E’, as an estimate of left atrial pressure [9], was similar in the presence or absence of LV-WMA, and was not changed after DSE test, except at the inferior region where it decreased after stress. These findings may reflect similar preload in both groups but with better LV relaxation in the absence of WMA.

Early diastolic global RV filling peak E-wave velocity increased significantly after DSE test and was similar in both groups at rest and after stress. Regional RV early filling E’-wave velocity by tissue Doppler was similar in both groups and did not change after stress. The RV E/E’ ratio, an indicator of right atrial pressure [10], mildly increased after DSE test the normal group, but was similar in both groups at rest and after DSE test. Thus, it seems that relaxation of RV was similar in both groups and was not affected by the presence of LV-WMA, both at rest and after DSE test. Late diastolic tricuspid A-wave velocity increased in both groups after DSE test, but at rest it was higher in the normal group than in the abnormal group.

It is expected to find an increase in late diastolic A-wave mitral velocity in association with the increase in heart rate with dobuatmine as previously reported [11]. The increase in diastolic and systolic pulmonary venous velocities after DSE test in the normal group is an indicator of an increase in stroke volume in these subjects. Previously, it was reported that maximal stroke volume is achieved at a dobutamine infusion dose of 20 μg/kg/min and tends to decline at higher doses in subjects without LV-WMA [12]. As stroke volume increases with dobutamine, it is expected to find an increase in early diastolic E-wave velocity as we found in normal subjects. The elimination of increase in pulmonary venous velocities and in E-wave mitral velocities with dobutamine in subjects with LV-WMA may reflect abnormality in LV relaxation and consequently absence of increase in stroke volume in subjects with coronary artery disease. Thus, despite the similarity of global early diastolic LV filling in both groups at rest, LV relaxation reserve is abolished in subjects with coronary artery disease and LV-WMA.

While global LV filling was similar at rest in both groups, regional early diastolic LV filling by tissue Doppler was more sensitive and distinguished the normal group by having faster early diastolic E-wave tissue Doppler velocities. This difference in regional early diastolic velocities was translated into the better global early diastolic LV filling reserve in normal group. Similar to global LV late diastolic A-wave velocity, the increase in heart rate with dobuatmine resulted in higher regional tissue Doppler late diastolic A-wave velocities in both groups irrespective of the presence of LV-WMA. After DSE test, neither E or A regional tissue Doppler velocities, nor the ration of E/E’ or A/A’ distinguished between the groups.

The presence of LV-WMA did not affect RV early diastolic filling. Thus, with the increase in stroke volume after dobuatmine, E-wave tricuspid valve velocities increased in both groups. Tricuspid valve early diastolic E-wave velocity was similar in both groups both at rest and after stress. Higher heart rates with dobuatmine, as expected, increased A-wave tricuspid valve velocity without significant intergroup difference. Regional early diastolic E-wave velocity and E/E’ ratio were similar in both groups and did not change with dobutamine.

The structure of the myocardium of RV and LV is somewhat different. Thus, contrary to the LV, which is composed of three myocardial fiber layers - superficial obliquely directed fibers, middle circumferentially directed fibers and deep inner layer with longitudinal fibers, the RV wall is composed of two myofiber layers [13]. The direction of RV myocardial fibers of the superficial layer is circumferential parallel to the atrioventricular groove, turns oblique toward the apex, and is continuous with the superficial layer of the left ventricle [14], while the inner myocardial layer of the RV is longitudinally directed from base to apex. In addition to the pericardium, and the sharing of the ventricular septum, this structural myocardial continuity between the ventricles is one of the causes of ventricular interdependence. It was reasonable to expect some effect of LV-WMA on RV filling. The absence of such effect highlights the superiority of diastolic properties of the RV.

### Conclusions

Global LV relaxation as evaluated by early diastolic global LV filling parameters was not affected by LV-WMA at rest; however, LV-WMA abolished their reserve. RV E-wave velocities increased after DSE test, and were not affected by LV-WMA. LV-WMA reduced regional LV annular E’ velocities at rest but did not affect their reserve. Late diastolic A-wave velocities were not affected by WMA and increased after DSE test.

### Limitations

At high heart rates, E and A waves through the atrioventricular valves merge together and it is not possible to identify these waves separately. Therefore, these waves were recorded when the heart rate decreased below 110 bpm [7]. Tissue Doppler diastolic waves were more easily identified and discernible at higher heart rates, as reported previously [7]. Mitral M-mode color Doppler slopes were easily determined at higher heart rates.
